# The Longer the Storage Time, the Higher the Price, the Better the Quality? A ^1^H-NMR Based Metabolomic Investigation of Aged Ya’an Tibetan Tea (*Camellia sinensis*)

**DOI:** 10.3390/foods11192986

**Published:** 2022-09-25

**Authors:** Chenglin Zhu, Zhibo Yang, Li He, Xuan Lu, Junni Tang, Luca Laghi

**Affiliations:** 1 College of Food Science and Technology, Southwest Minzu University, Chengdu 610041, China; 2 College of Food Science, Sichuan Agricultural University, Ya’an 625014, China; 3 Department of Agricultural and Food Sciences, University of Bologna, 47521 Cesena, Italy

**Keywords:** Ya’an Tibetan tea, metabolome, proton nuclear magnetic resonance spectroscopy, ageing time

## Abstract

As an essential beverage beneficial for Tibetan people, Ya’an Tibetan tea has received scarce attention, particularly from the point of view of the characterization of its metabolome. The aim of the study is to systematically characterize the metabolome of Tibetan tea by means of untargeted ^1^H-NMR. Moreover, the variations of its metabolome along ageing time are evaluated by taking advantage of univariate and multivariate analyses. A total of 45 molecules are unambiguously identified and quantified, comprising amino acids, peptides and analogues, carbohydrates and derivates, organic acids and derivates, nucleosides, nucleotides and catechins. The concentrations of amino acids, organic acids, carbohydrates and catechins are mainly determined by ageing time. The present study would serve as a reference guide for further work on the Ya’an Tibetan tea metabolome, therefore contributing to the related industries.

## 1. Introduction

Tea is regarded as one of the most popular and widely consumed beverages throughout the world [1]. The consumption of tea has increased yearly, not only due to the distinct flavor and pleasant taste, but also to the important physiological state and potential health benefits, granted by the presence of various compounds, for instance, carbohydrates, polyphenols, caffeine, amino acids, vitamins and purine alkaloids [2]. There are five main marketed varieties of tea, differentiated by their fermentation process. In detail, green tea is unfermented, white tea is lightly fermented, oolong tea is partially fermented, black tea is fully fermented and dark tea is post-fermented. Among them, dark tea is a unique post-fermented tea produced by pile fermentation attributed to microbial fermentation [3], whose history could be dated back to the Ming Dynasty around 1500 A.D. [4]. In the dark tea family, it is worth mentioning that Ya’an Tibetan tea was initially produced in Southwestern China and then carried via the mountains to Tibet [5], where it has become an essential beverage benefitting millions of Tibetan people.

The Tibetan Plateau is not well suited for cultivating vegetables, fruit and trees, due to its altitude between 3000 and 5000 m. Thus, highly caloric foods are typically consumed, with low fiber intake, by Tibetan people in daily life [6]. These high-protein, high-lipid diets can effectively help them overcome the harsh environment, while they may also increase the risk of cardiovascular and indigestion diseases. Interestingly, they generally drink Tibetan tea along with meals based on high fat milk and red meat, therefore balancing cholesterol and fat absorption. Till now, numerous studies have demonstrated that Ya’an Tibetan tea exhibits antioxidant, cytoprotective [7] and antiradiation effects [8] by in vitro and in vivo experiments. Moreover, Li et al. reported that Ya’an Tibetan tea can effectively lower blood pressure, remove blood lipids and reduce the generation of atherosclerosis [9], which could be linked to its good inhibitory effects on lipase [10]. Intake of Ya’an Tibetan tea is also confirmed to have an anti-inflammatory effect through regulating gut microbiota and altering inflammation and immune system pathways expression in mice models [11]. 

Untargeted metabolomics, which could provide holistic information about a biofluid, is regarded as the most comprehensive representation of an organism’s phenotype [12]. This approach attempts to provide qualitative and quantitative information of low weight metabolites (<900 Da) from biological samples. Until now, metabolomics has been widely applied to investigate tea metabolome profiles altered by internal and external factors, such as fermentation process [13,14], shade treatment [15] and seasonal variation [16]. As one of the mostly applied techniques for metabolomics investigations, ^1^H-NMR spectroscopy has been applied in related fields thanks to its non-invasive nature, highly reproducible molecules’ quantification and effectiveness in analyzing a diverse range of compounds. Lee et al. evaluated strong inter-country and inter-city relationships in the quantities of theanine and catechin derivatives found in green and white teas by means of ^1^H-NMR [17]. Ohno et al. found that growing tea at higher altitudes leads to a high amount of theanine and caffeine and to low levels of thearubigins, especially thearubigin 3,3′-digallate [18].

Commonly, consumers perceive a direct connection between storage time and quality, accepting therefore higher prices for more aged teas. However, there have been no studies on the change of comprehensive metabolomic profiles during storage for Ya’an Tibetan tea, except for one paper published recently which just referred to concentrations of polyphenols and catechin compounds affected by storage time [19]. Moreover, there is limited knowledge of expected concentrations of metabolites from Ya’an Tibetan tea. To fill these gaps, the present study aims to systematically characterize the metabolomic profiles of Ya’an Tibetan tea by means of ^1^H-NMR. Furthermore, the evolution of the metabolomic profile of Ya’an Tibetan tea along storage time was evaluated. This study could offer guidance for consumers to select Ya’an Tibetan tea products and act as a reference for the related industries to produce high-quality products. 

## 2. Materials and Methods

### 2.1. Sampling

As described by Xie et al., five years could be considered as the milestone from the perspective of chemical constituents of Pu-erh tea, a fully fermented tea similar to Tibetan teas [20]. For this reason, we collected Ya’an Tibetan tea samples spanning 10 years of ageing. All the Ya’an Tibetan tea samples were purchased from Sichuan Ya’an Tea Factory Co., Ltd. (Sichuan, China). The samples comprised 30 Tibetan tea samples, sorted into three groups, namely 1 year, 5 years and 10 years. Each group included ten samples.

### 2.2. Metabolome Analysis

By adapting the procedure described by Ohno et al. [18], one gram of each Ya’an Tibetan tea was added to 30 mL of boiling bi-distilled water. The mixture was kept for 4 min at 95 °C and then left at room temperature for 10 min. After vortex mixing for 1 min and centrifuging for 15 min at 14,000 rpm and 4 °C, 0.5 mL of supernatant were taken to a new Eppendorf tube, and then 0.2 mL of a D_2_O solution of 3-(trimethylsilyl)-propionic-2,2,3,3-d_4_ acid sodium salt (TSP) 10 mM was added, used as NMR chemical-shift reference, buffered at pH 7.00 ± 0.02 using 1 mol/L phosphate buffer. In order to avoid microbial proliferation, 10 μL of NaN_3_ 2 mmol/L was also added. Finally, each sample was centrifuged again at the above conditions. The workflow of sample preparation procedure is shown in Figure S9a. 

^1^H-NMR spectra were performed at 298 K equipment with an AVANCE III spectrometer (Bruker, Milan, Italy) operating at a frequency of 600.13 MHz. Taking advantage of presaturation, the HOD residual signal was suppressed. This was done by employing the noesygppr1d sequence, part of the standard pulse sequence library. Each spectrum was acquired by summing up 256 transients by means of 32 K data points over a 7184 Hz spectral window, with an acquisition time of 2.28 s and a recycle delay of 5 s. The workflow of spectra processing is shown in Figure S9b. In detail, ^1^H-NMR spectra baseline-adjusted through peak detection in accordance with the “rolling ball” principle [21] implemented in the baseline R package [22]. Differences in water and fiber content among samples were calculated by means of probabilistic quotient normalization (PQN) [23] applied to the entire spectra array. The signals were assigned by comparing their chemical shift and multiplicity to Chenomx software library (Chenomx Inc., Edmonton, AB, Canada, v.8.4) and authentic material or published data [18]. Integration of the signals was obtained for each molecule using rectangular integration.

### 2.3. Statistical Analysis

Statistical analysis was performed in R computational language [24] and online metabolomic data analysis platform MetaboAnalyst (https://www.metaboanalyst.ca, Montreal, QC, Canada, v.5.0, accessed on 25 July 2022). Prior to univariate analysis, concentrations of molecules in each group that were not-normally distributed were transformed in accordance with Box and Cox [25]. And then, to figure out perturbations caused to single molecules by the effects considered, *t*-tests were performed with a cut-off *p* value below 0.05. 

## 3. Results

### 3.1. ^1^H-NMR Spectra of Ya’an Tibetan Tea Samples

**Figure 1:**
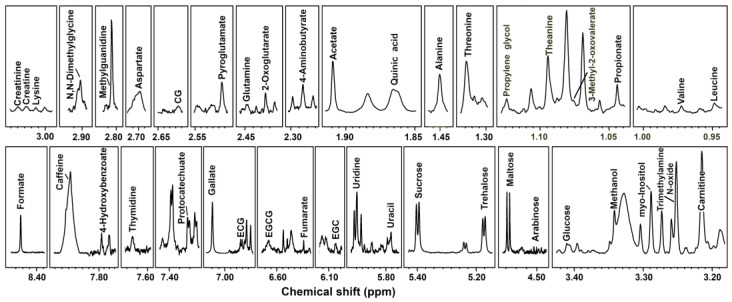
Portions of ^1^H-NMR spectra from typical Ya’an Tibetan tea samples. Name of the molecules appears on the signals used for their quantification. The vertical scale of each portion is conveniently set to ease the signals observation.

Represented spectra from ^1^H-NMR were assigned as pictorially described in Figure 1, while the entire concentrations for each sample are reported in the supporting materials. An important step for signal assignments performed by ^1^H-NMR is the comparison with references of the fine structure of the signals visually [26]; there are supplemental material reports, for each characterized molecule, and superimpositions of spectra registered and simulated for pure compounds (Supplementary Materials, Figures S1−S8). In addition, to increase the reproducibility of our results, the functional groups and ppm for each identified metabolite are reported in Table 1. 

### 3.2. Ya’an Tibetan Tea Metabolome Variations along Storage Time

**Figure 2:**
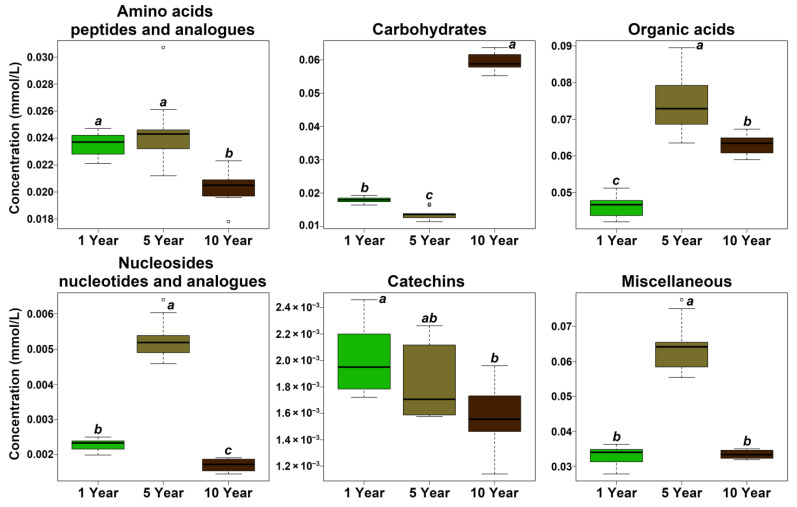
Concentrations of the main classes of molecules quantified by ^1^H-NMR among the three groups. The italic lowercase letters above each box indicated the significances of the comparisons among the three groups, where a common superscript is not significantly different.

**Figure 3:**
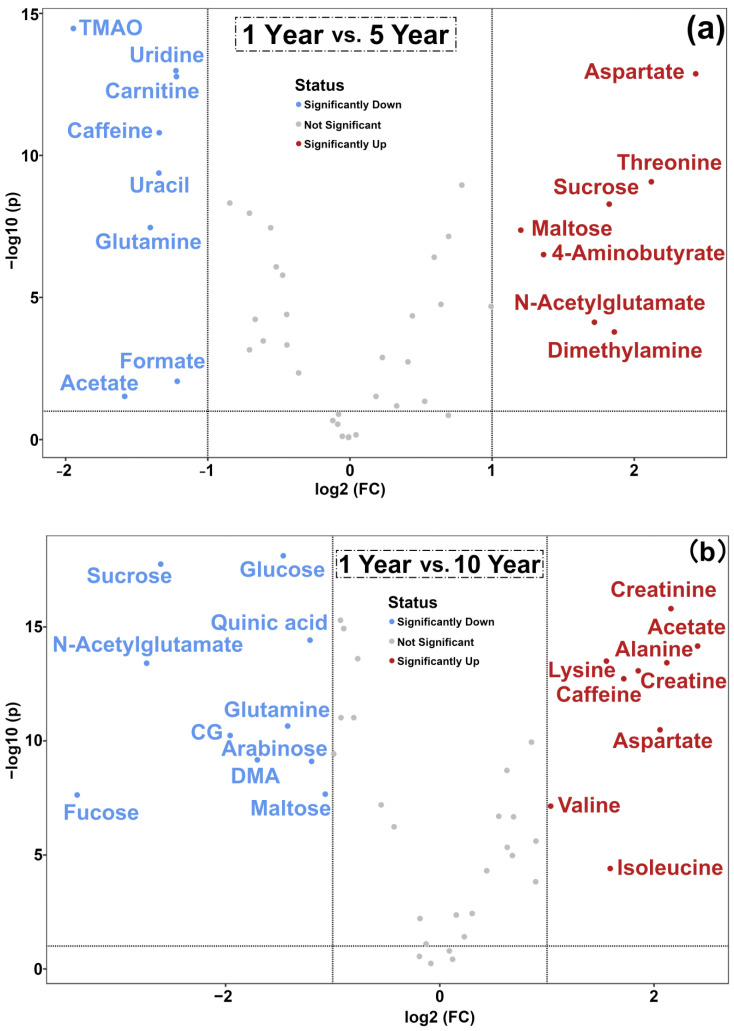

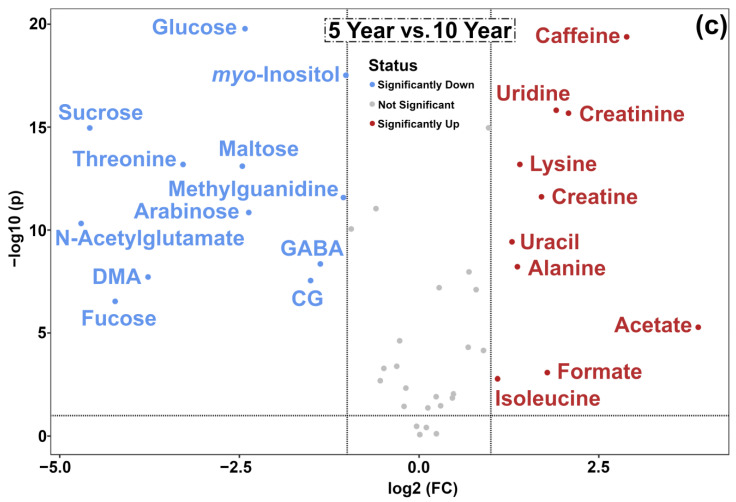
Volcano plot built on the concentration of molecules in each of the two groups. (**a**) indicates 1 Year vs. 5 Year, (**b**) indicates 1 Year vs. 10 Year and (**c**) indicates 5 Year vs. 10 Year.

**Figure 4:**
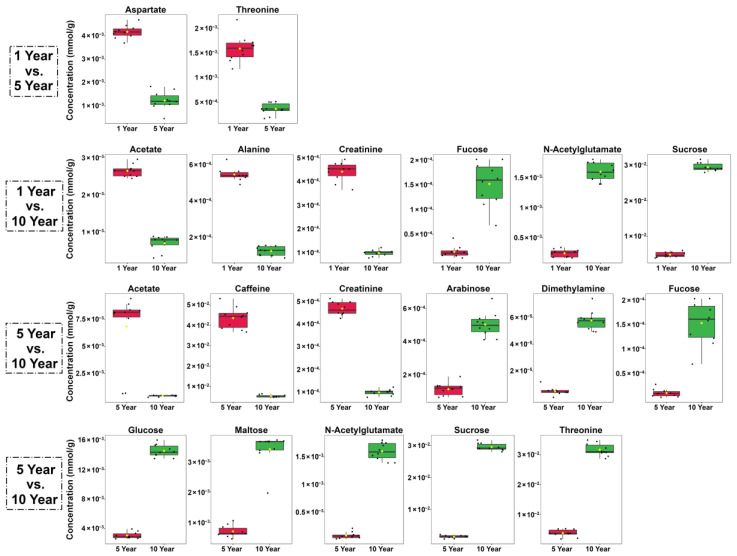
Boxplot based on the concentration of molecules whose fold change is above 2 in each of the two groups. Asterix indicates the mean value of the group in each box.

As can be seen from Table 1, a total of 45 molecules were identified and quantified, mainly pertaining to the chemical groups of amino acids, peptides and analogues (16), carbohydrates (6), organic acids (10), nucleosides, nucleotides and analogues (3), catechins (3) and miscellaneous (7). As shown in Figure 2, the concentration of total amino acids, peptides and analogues was significantly reduced from 5 years to 10 years. Moreover, the content of total carbohydrates was reduced in the early storage period, and then the content increased markedly as storage was prolonged. As for organic acids, nucleosides, nucleotides and analogues, and miscellaneous, their trends were similar, namely, increasing first until five years and decreasing afterwards. The amount of catechins was significantly decreased after 5 years. However, it is worthy to note that several represented molecules did not exactly follow the same trend as their groups. Therefore, volcano plots, reported in Figure 3, evidence the main differences between each couple of time points. Moreover, concentrations of molecules showing a fold change above 2 in each of the two groups are shown as boxplot, in Figure 4. 

Comparing the first five years with the subsequent five, the concentration of several molecules exhibited opposite trends, namely maltose, n-acetylglutamate, dimethylamine, 4-aminobutyrate, caffeine, uridine, uracil, formate and acetate, as shown in Figure 3a,c and Figure 4. However, widening the view to comprise the entire period evaluated (Figure 3b), the amounts of creatinine, alanine, lysine, acetate, caffeine and isoleucine were significantly increased along storage time, while the levels of sucrose, glucose, n-acettylglutamate, CG, dimethylamine, fucose, arabinose and maltose appeared as significantly decreased.

**Figure 5:**
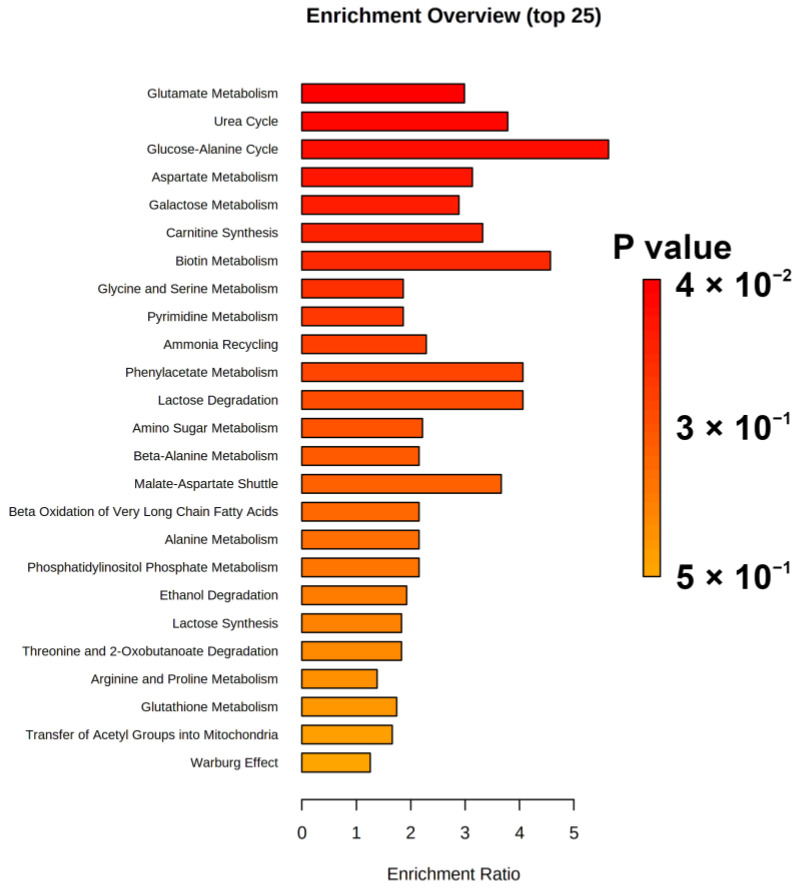
Enrichment analysis built on the concentration of molecules significantly varied along storage time.

To obtain deeper details into which metabolic pathway could undergo the widest modifications with storage time, an enrichment analysis was performed by means of the MetaboAnalyst platform. The pathways evidenced as potentially altered by storage time (*p*-value < 0.05) were glutamate metabolism, urea cycle and glucose-alanine cycle, as shown in Figure 5. 

## 4. Discussion

As one of the essential beverages for Tibetan people, most of the works dealing with Tibetan tea have been focused on its beneficial properties for human health and on safety risk assessments. For instance, Li et al., found that high doses (400 mg/Kg/d) of Tibetan tea supplementation reduced bodyweight gains and markedly attenuated serum lipid profiles and atherosclerosis index in mice model [9]. Xie et al. evaluated that Tibetan tea has antioxidative or cytoprotective properties linked to phenolic compounds, such as gallic acid and four catechins (catechin, CG, ECG and EGCG) [7]. Ye et al. assessed ten mycotoxins in Tibetan tea samples, ruling out potential risks for consumers [27]. In contrast, less attention has been devoted to Tibetan tea itself, with only a few papers having attempted to investigate the volatile [28] and phenolic compounds [7] in Tibetan tea by means of metabolomic approaches. To the best of our knowledge, there are no complete reports about quantitative information for each molecule that can be characterized by ^1^H-NMR. Moreover, there seem to be no reports about the variations of Ya’an Tibetan tea metabolomic profiles with ageing time. To gain more information about these aspects, the present work attempts, for the first time, to provide reference quantitative values for the molecules mostly present in the Ya’an Tibetan tea metabolome, as observable by ^1^H-NMR. A total of 45 metabolites were unambiguously characterized, a number much higher than those previously obtained based on the same platform [15,17,18]. The quantified molecules mainly pertained to the categories of amino acids, peptides and analogues, carbohydrates and derivates, organic acids and derivates, nucleosides, nucleotides and analogues, catechins and miscellaneous. The most important chemical constituents that influence the taste and flavor of tea infusions are sugars, organic acids, amino acids, polyphenols, caffeine, flavonols and volatile flavor compounds [29]. In the present work, we found that the concentrations of 12 amino acids, 3 organic acids, 6 sugars, 2 nucleosides, nucleotides and analogues, and 5 miscellaneous in total were significantly altered with ageing time by means of volcano plot. According to the above observations, we could infer that ageing time would eminently affect the taste and flavor of Ya’an Tibetan tea. 

In terms of amino acids, several remarkable works have indicated that there is a relationship between the quality of tea and the amino acid contents [30], with consequences on fresh and brisk tastes of tea infusion and aroma substances [31,32]. Moreover, Alcázar et al. observed a clear relation between the amino acids content and the elaboration process of teas. In detail, unfermented or lightly fermented teas exhibit higher levels of free amino acids than fully fermented or post fermented ones [1]. Focusing on the total amino acids content, the present work noticed no significant variations but a slightly increase in the first five years of ageing, while a significant decrease occurs in the following five years. Such a phenomenon could be linked to the degradation of proteins into amino acids during the early stage of pile-fermentation process [33], and then part of amino acids could evolve into volatile compounds along storage time [34]. Among the amino acids quantified, it is worth noticing that theanine, a unique amino acid that is found almost exclusively in tea, could contribute to the brothy sweet umami taste of tea [35,36]. Cheng et al. found that theanine content was reduced by 93.51% during Qingzhuan tea processing [37]. Our results were in line with such trends, with the fermentation procedure reducing the contents of theanine, even if to lesser extents. This could be due to the distinct fermenting conditions and, in turn, to the different microbial community.

As one of the primary inhibitory neurotransmitters, 4-aminobutyrate plays an important role in the vertebrate central nervous system and has antianxiety and antihypertensive effects [38]. 4-aminobutyrate is mainly biosynthesized through the irreversible α-decarboxylation of Glutamate to 4-aminobutyrate, which is catalyzed by pyridoxal 5′-phosphate (PLP)-dependent glutamate decarboxylase (GAD) in plants [39]. Even if glutamate could not be quantified in the present study, this biosynthetic route for 4-aminobutyrate may be confirmed by the trend we highlighted for glutamine. In fact, glutamine is synthesized through glutamine synthetase from glutamate, and its concentration shows a trend opposite to that of 4-aminobutyrate. Such a pathway was highlighted by enrichment analysis, too, further indicating that it plays an important role during the pile-fermentation process of Ya’an Tibetan tea. 

Organic acids are considered as major detrimental contributors to overall taste of dark teas. In terms of total organic acids, their content was significantly increased in the first five years, while decreased during the following five years. Therefore, we could speculate that five years could be regarded as the line of demarcation during pile-fermentation [20]. Among the characterized organic acids, acetate is produced by acetic acid bacteria from glucose. In the present study, the concentration of acetate increased in the first five years, followed by a decrease in the next five years. Such result was in line with previous studies on the topic [20,40]. Gallate is another important compound widely found in tea leaves, which could be regarded as a precursor for catechin catabolism. Gallate is derived from the hydrolysis of procyanidins and gallyolated catechins and degraded into methoxy phenolic compounds during dark tea processing [41]. The trend of gallate we found is in agreement with Qingzhuan tea process [37], but opposite that found for Pu-erh tea [42]. This discrepancy is probably due to the degradation of gallate, which exceeds the hydrolysis of gallyolated catechins during Ya’an Tibetan tea processing. 

Catechins, which account for 60−80% of tea polyphenols, are the main components contributing to the antioxidant activity of tea [4]. Catechins, together with caffeine [43] and volatile components [44], have also been used to differentiate tea categories. The contents of catechins in dark teas share the same trends with amino acids, with a concentration much lower than the one characterizing unfermented and semi-fermented teas [4]. In the present study, the overall trend of catechins was in agreement with the previous work, while a smaller number of catechins were quantified, which could be linked to the discrepancy of detection sensitivity between different metabolomic approaches, namely ^1^H-NMR and UPLC-QqQ-MS/MS [19]. Such an observation could be explained considering that post fermentation process highly decreases the contents of catechins and form pigments such as theabrownines, which have been suggested as linked to the oxidation and condensation of catechins during post fermentation by microorganisms [42,45]. As catechins contribute to the astringency taste of tea, their decrease with storage could lead to a decline in the astringency taste and could deepen the color of Ya’an Tibetan tea infusion. Together with a significant increase in the total content of carbohydrates, prolonging storage time may have beneficial effects on the improvement of tea infusions’ sensory evaluation.

## 5. Conclusions

To the best of our knowledge, the present study, for the first time, has been devoted to obtaining a holistic metabolomic representation of Ya’an Tibetan tea, by providing quantitative information of Ya’an Tibetan tea metabolome through untargeted ^1^H-NMR. A remarkably higher number of metabolites than previously reported was characterized by a single platform. The contents of amino acids, organic acids, carbohydrates and catechins are mainly determined by ageing time, which would eminently affect the taste and flavor of Ya’an Tibetan tea. As we purchased Ya’an Tibetan tea samples from the same factory, the conditions of fermentation could be considered as identical across the samples analyzed, but there are still several factors (such as the variations in raw tea leaves collected each year) that should be taken into consideration for further investigations. The present study could serve as a reference guide for further Ya’an Tibetan tea metabolome studies.

## Figures and Tables

**Table 1:** Information for molecules identification by means of ^1^H-NMR.

	ppm	Functional Group	Multiplicity *
**Amino acids, Peptides and Analogues**			
4-Aminobutyrate	2.2854	CH_2_-2	t
Alanine	1.4754	CH_3_	d
Aspartate	2.7009	CH_2_	dd
Carnitine	3.2146	CH_3_	s
Creatine	3.0270	CH_3_	s
Creatinine	3.0405	CH_3_	s
Glutamine	2.4492	CH_2_-2	m
Isoleucine	0.9906	CH_3_-4	d
Leucine	0.9479	CH_3_	t
Lysine	3.0130	CH_2_	t
N,N-Dimethylglycine	2.8999	CH_3_	s
N-Acetylglutamate	2.2318	CH_2_-2	t
Pyroglutamate	2.5275	CH_2_-5	m
Threonine	1.3117	CH_3_	d
Theanine	1.0936	CH_2_	m
Valine	0.9718	CH_3_	d
**Carbohydrates**			
Arabinose	4.5082	CH_2_	d
Fucose	1.2313	CH_3_	d
Glucose	3.4074	CH-3	t
Maltose	4.6291	CH	d
Sucrose	5.3954	CH	d
Trehalose	5.1807	CH	d
**Organic Acids**			
2-Oxoglutarate	2.4246	CH_2_-2	t
3-Methyl-2-oxovalerate	1.1004	CH_3_-4	d
4-Hydroxybenzoate	7.7896	CH_2_-3	d
Acetate	1.9082	CH_3_	s
Formate	8.4454	CH	s
Fumarate	6.5080	CH	s
Gallate	7.0203	CH	s
Propionate	1.0438	CH_3_	t
Protocatechuate	7.3737	CH	dd
Quinic acid	1.8642	CH_2_	d
**Nucleosides, Nucleotides and Analogues**			
Thymidine	7.6287	CH-7	s
Uracil	5.7969	CH-6	d
Uridine	5.8970	CH	s
**Catechins**			
Catechin gallate (CG)	2.6328	CH_2_	dd
Epicatechin gallate (ECG)	6.9439	CH	d
Epigallocatechin gallate (EGCG)	6.6304	CH	d
**Miscellaneous**			
Caffeine	7.8612	CH-9	s
Dimethylamine	2.7132	CH_3_	s
Methanol	3.3495	CH_3_	s
Methylguanidine	2.8057	CH_3_	s
*myo*-Inositol	3.2878	CH	t
Propylene glycol	1.1248	CH_3_	d
Trimethylamine N-oxide	3.2494	CH_3_	s

* s stands for singlet, d stands for doublet, t stands for triplet, and m stands for multiplicity.

## Data Availability

Data is contained within the article or supplementary material.

## Supplementary Materials

The following are available online at https://www.mdpi.com/article/10.3390/foods11192986/s1, Figure S1–S8: Molecules assignment and quantification; Figure S9: Sample preparation and spectra processing procedures for ^1^H-NMR.
