# Supplementary material for: The Longer the Storage Time, the Higher the Price, the Better the Quality? A 1H-NMR Based Metabolomic Investigation of Aged Ya’an Tibetan Tea (Camellia sinensis)
Source: Foods. 2022 Sep 25;11(19):2986. doi: 10.3390/foods11192986 (PMC9563412; doi:10.3390/foods11192986)

The longer the storage time, the higher the price, the better the quality? A ^{1}H-NMR based metabolomic investigation of aged Ya'an Tibetan tea (*Camellia sinensis*)

Chenglin Zhu[1], Zhibo Yang[1], Li He[2], Xuan Lu[1], Junni Tang[1,*] and Luca Laghi[3,*]

Molecules assignment and quantification

Figures S1-S8: Pictorial description of the molecules' assignment and quantification procedure by Chenomx software. Upper panel - portions of the spectra in white-washed mode. Lower panel – One representative registered spectrum (black line) superimposed to the signals simulated by Chenomx software (red line) for each of the molecules listed. The black and red dashed lines evidence the signals used for quantification purposes.

Figure S1

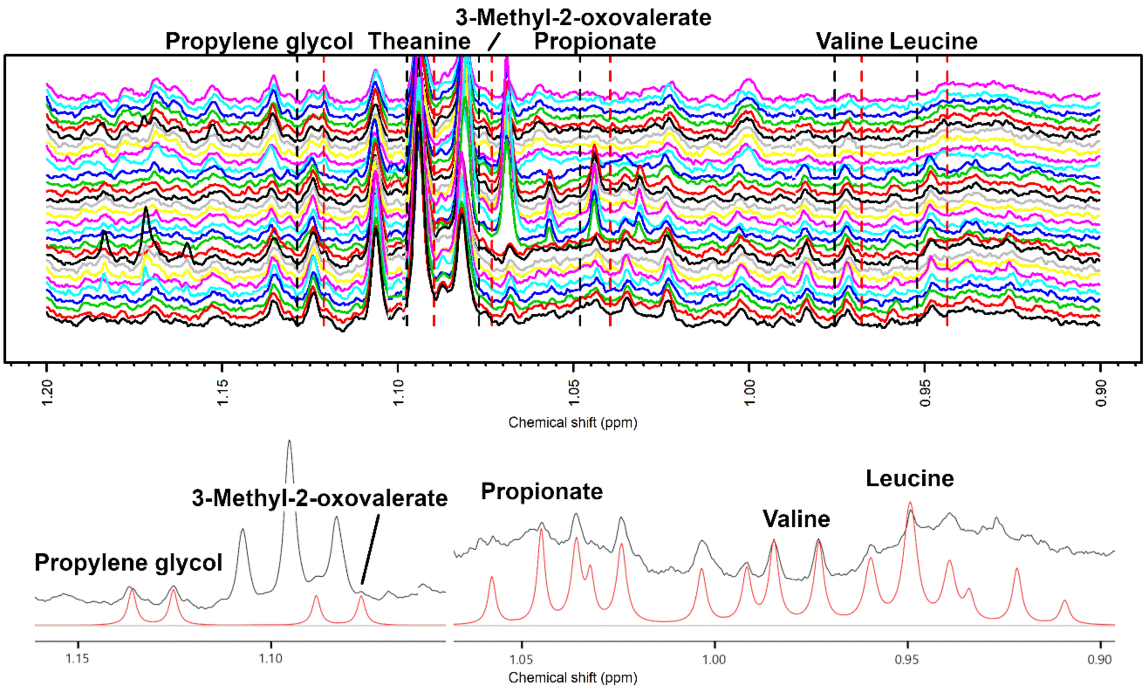

Figure S2

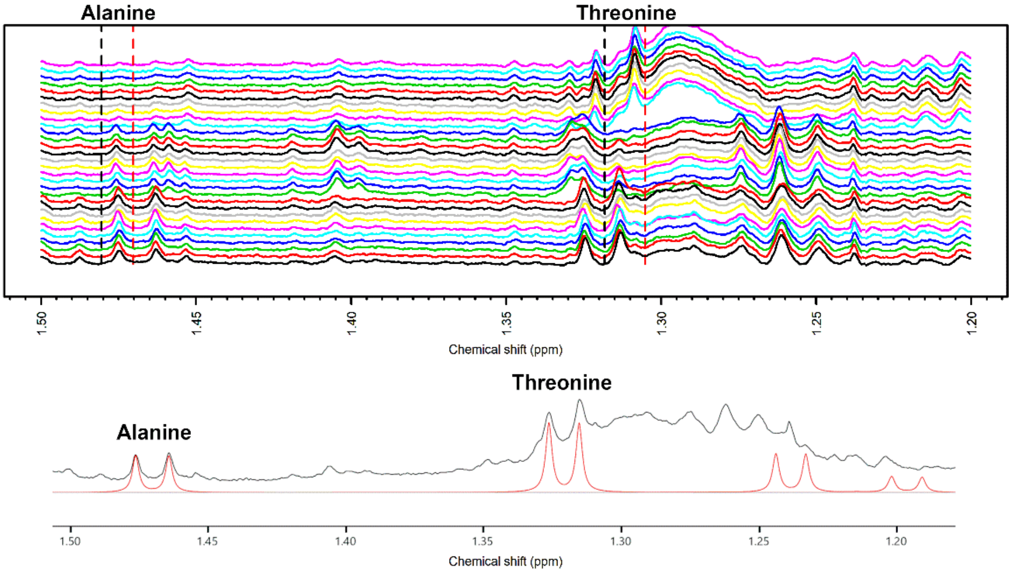

Figure S3

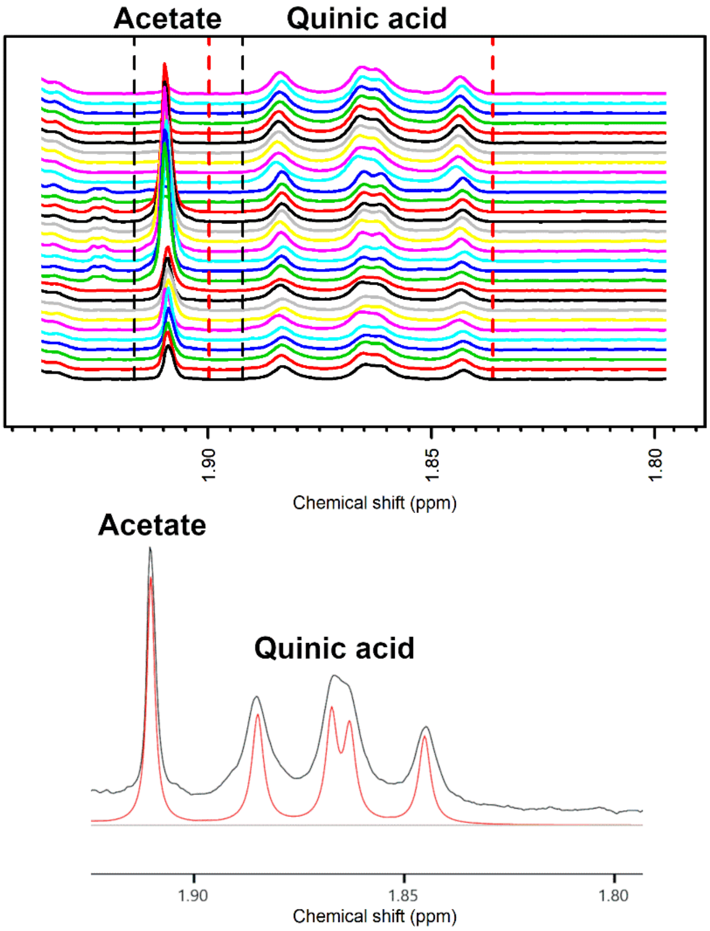

Figure S4

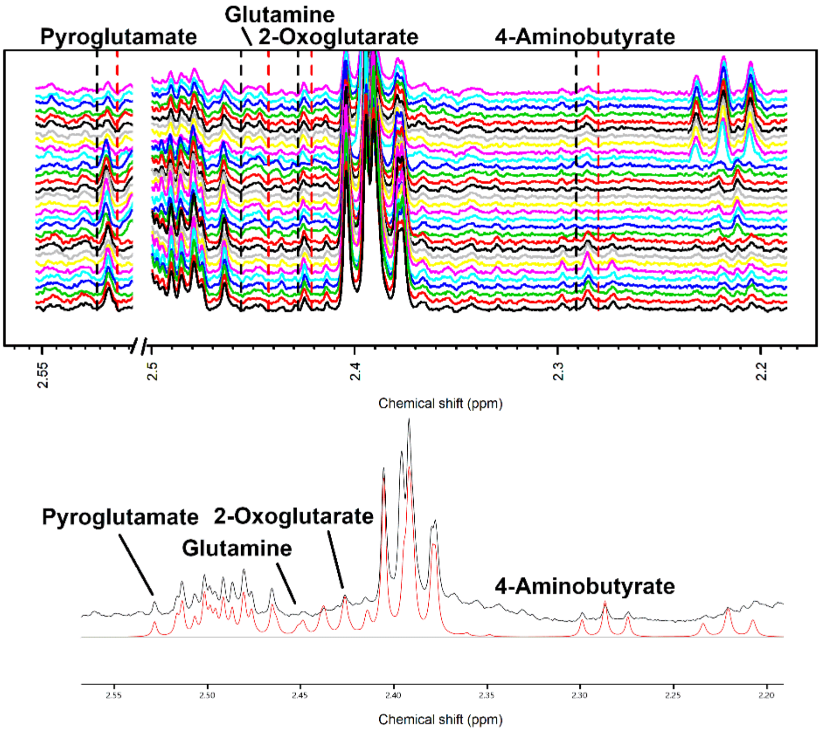

Figure S5

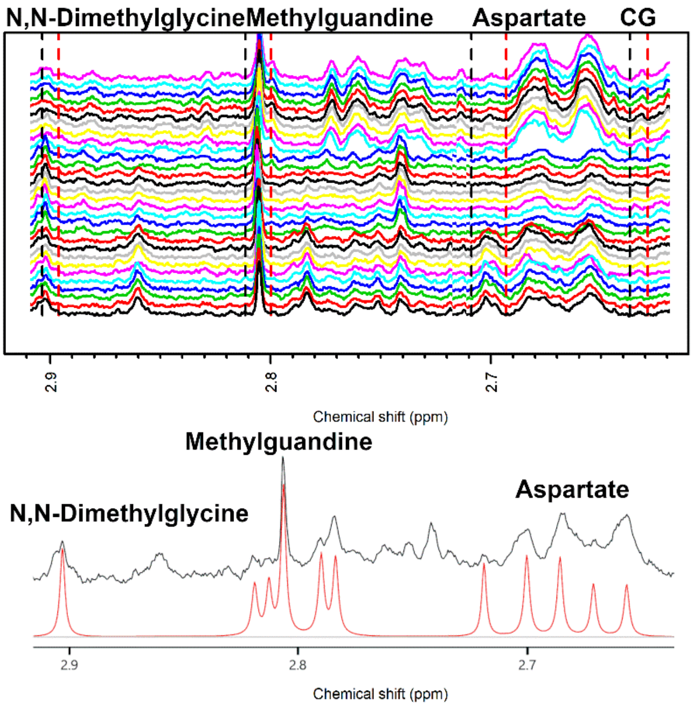

Figure S6

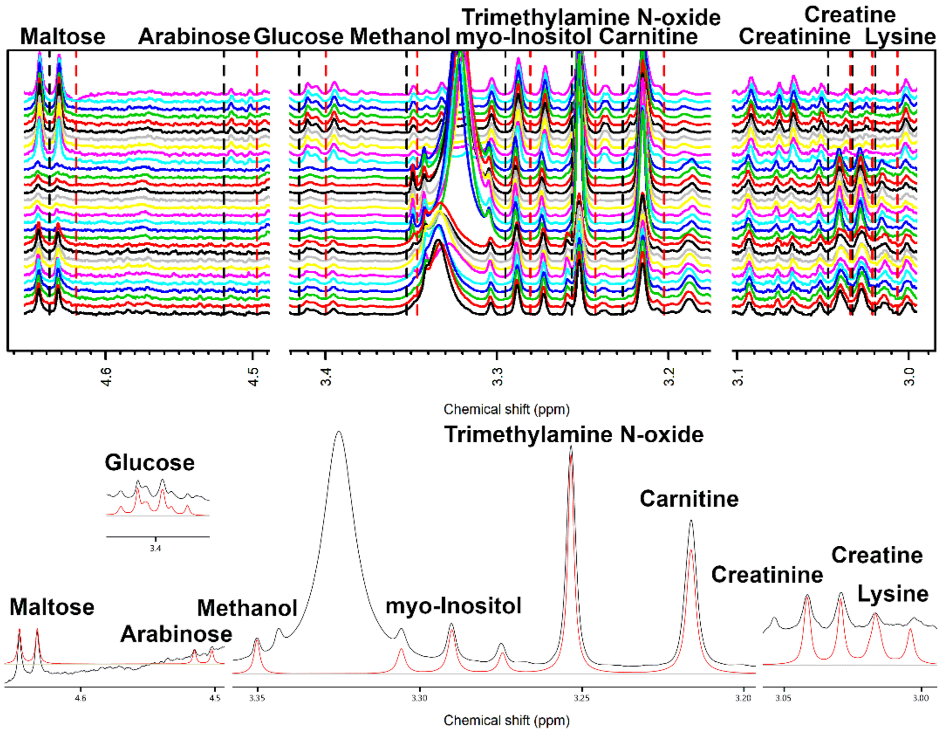

Figure S7

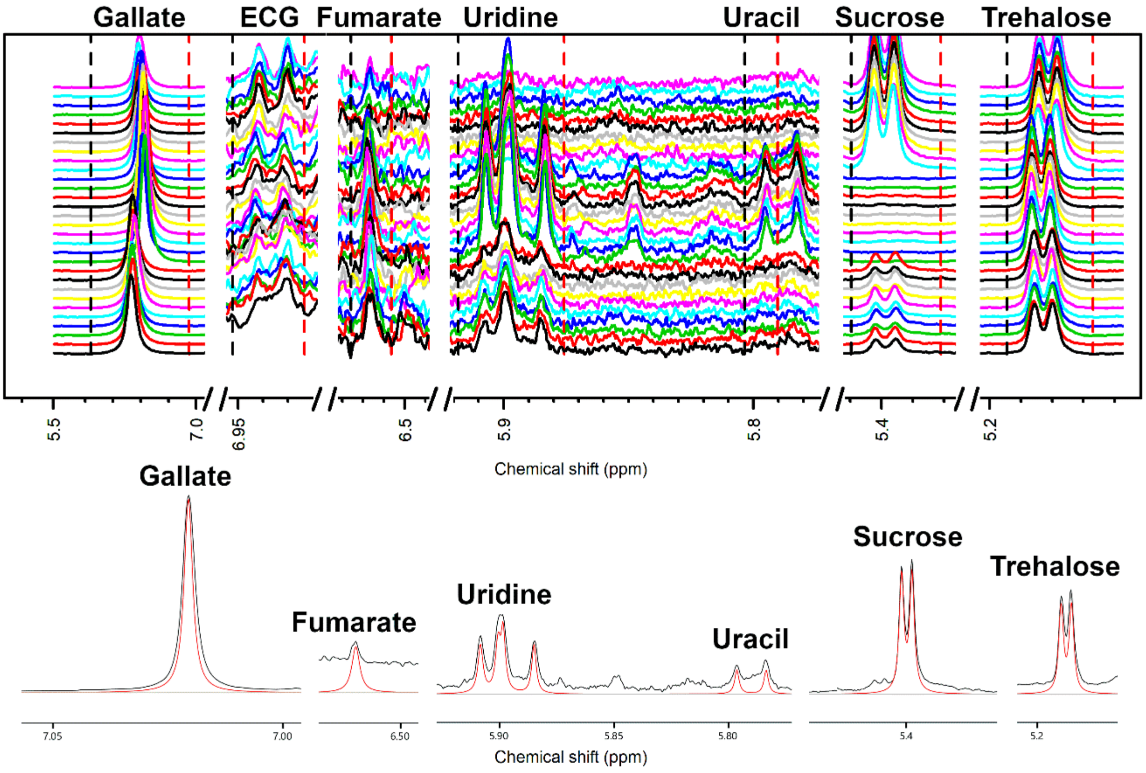

Figure S8

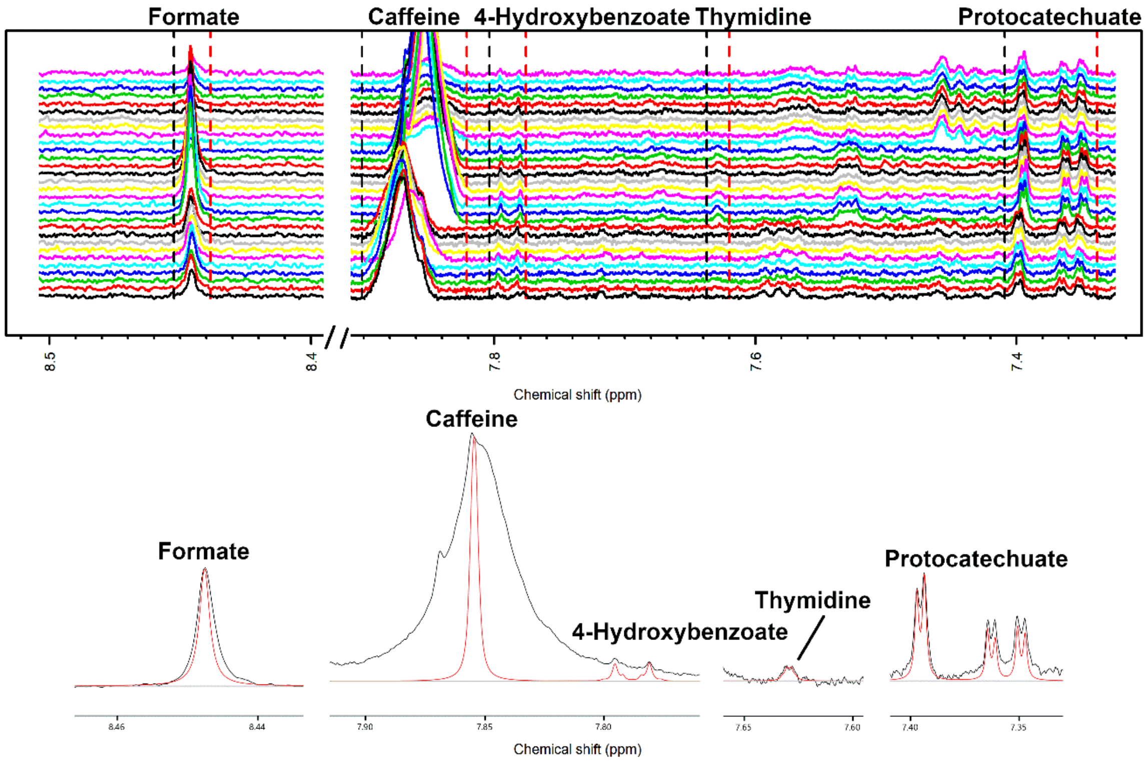
Formate
Caffeine
4-Hydroxybenzoate
Thymidine
Protocatechuate
8.5
8.4
7.8
7.6
7.4
Chemical shift (ppm)
Caffeine
Formate
Protocatechuate
Thymidine
4-Hydroxybenzoate
8.46
8.44
7.90
7.85
7.80
7.65
7.60
7.40
7.35
Chemical shift (ppm)

Figure S9 Sample preparation and spectra processing procedures for ^{1}H-NMR

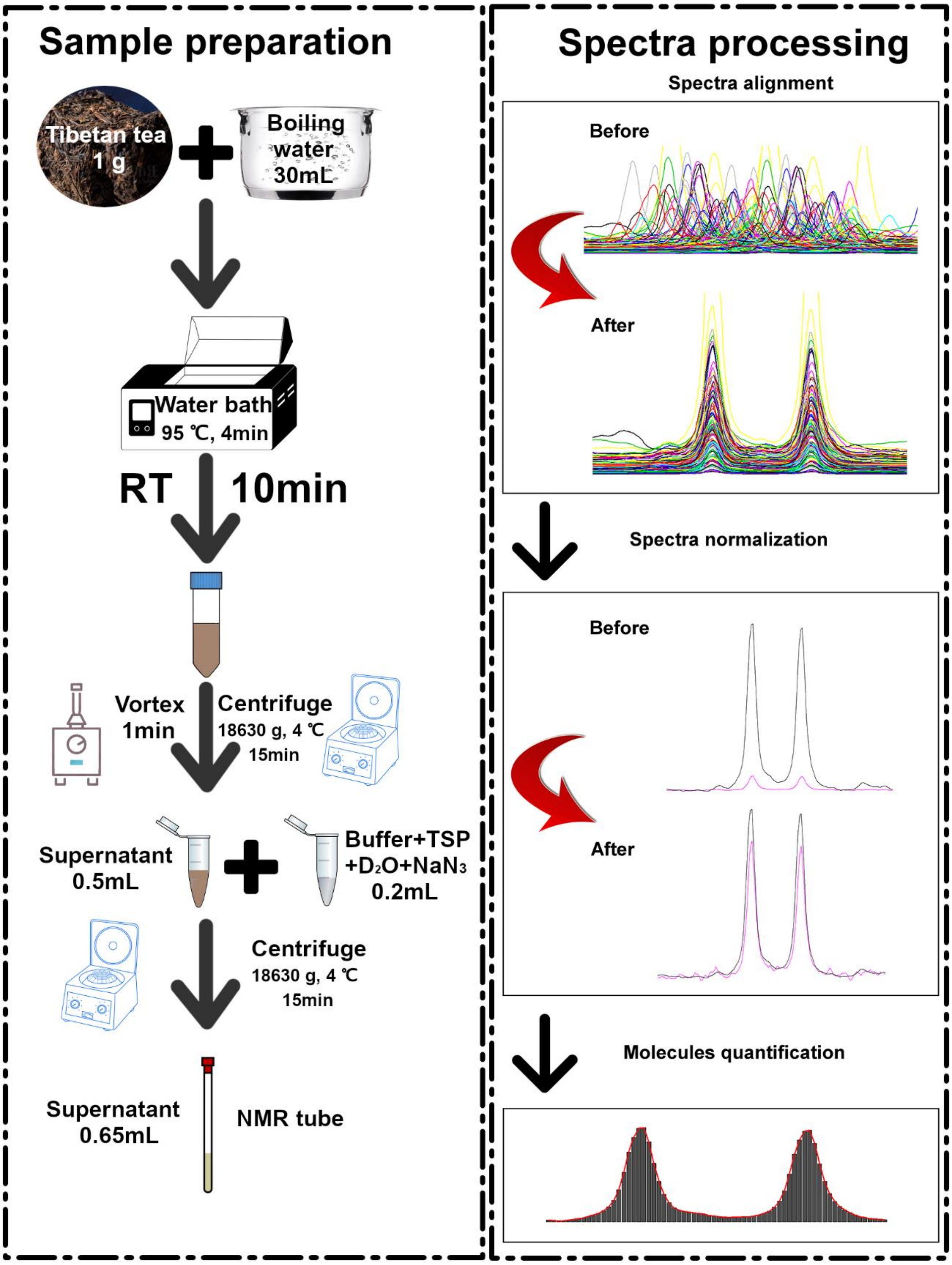

Supplement: Supplementary file 1 [file foods-11-02986-s001.zip › foods-1890937-supplementary.pdf]
